# Biomimetic Design and Extrusion-Based 3D Printing of TiO_2_ Filled Composite Sphere Scaffolds: Energy-Absorbing and Electromagnetic Properties

**DOI:** 10.3390/biomimetics10120804

**Published:** 2025-12-01

**Authors:** Marsel Akhmatnabiev, Alexander Petrov, Mikhail Timoshenko, Maxim Sychov, Semyon Diachenko, Maxim Arsentev, Alexander Bakulin, Ekaterina Skorb, Michael Nosonovsky

**Affiliations:** 1 Branch of B. P. Konstantinov Petersburg Nuclear Physics Institute of National Research Center “Kurchatov Institute”—I.V. Grebenshchikov Institute of Silicate Chemistry, St. Petersburg 199034, Russia; mahmatnabiev@spbti.ru (M.A.); timoshenkomv@vsk.sibur.ru (M.T.); 2 Scientific and Research Institute «Vector», St. Petersburg 197022, Russia; petrov_aa@nii-vektor.ru; 3 St. Petersburg State Technological Institute (Technical University), St. Petersburg 190013, Russia; msychov@spbti.ru (M.S.); svdiachenko@technolog.edu.ru (S.D.); 4 Central Research Institute of Structural Materials «PROMETEY», St. Petersburg 191015, Russia; 5 Infochemistry Scientific Center (ISC), ITMO University, 9 Lomonosova St., St. Petersburg 191002, Russia; arsentev_myu@list.ru (M.A.); 465113@niuitmo.ru (A.B.); skorb@itmo.ru (E.S.); 6 College of Engineering and Applied Science, University of Wisconsin-Milwaukee, Milwaukee, WI 53211, USA

**Keywords:** energy absorption, biomimetic cellular lattices, mechanical properties, cellular materials, antenna lens, dielectric permittivity, additive manufacturing, 3D printing, cellular gradient materials, triply periodic minimal surfaces

## Abstract

The development of composite materials with tunable dielectric properties that preserve mechanical performance is essential for next-generation radio engineering devices. In this study, composite filaments based on acrylonitrile–butadiene–styrene (ABS) with 0–40 wt.% TiO_2_ solid loading were developed for 3D printing. The dielectric permittivity and mechanical properties of the 3D-printed parts strongly depend on the TiO_2_ content. Using these filaments, we fabricated biomimetic lattices based on triply periodic minimal surfaces (TPMSs) using fused filament fabrication (FFF). The intrinsic porosity of the TPMS lattices further enables tuning of dielectric permittivity, facilitating their integration into gradient-index components. This multifunctionality was demonstrated by fabricating a spherical Luneburg lens prototype, which exhibited stable antenna performance in the 8.0–12.5 GHz frequency range. The results confirm that TPMS lattices based on the ABS-TiO_2_ composite can simultaneously deliver mechanical robustness and dielectric tunability, opening new pathways toward multifunctional components for advanced radio engineering systems and beyond.

## 1. Introduction

Modern microwave and radio engineering systems require compact, lightweight, and mechanically robust antennas capable of providing directive radiation and wide-angle scanning. The performance of these systems depends on the ability to control electromagnetic wave propagation through materials with graded dielectric properties. Among various beamforming devices, spherical dielectric lenses particularly the Luneburg lens (LL) [1] are considered one of the most promising solutions due to their capability to generate multiple radiation patterns and enable wide-angle scanning.

The classical Luneburg lens achieves this functionality through a continuous radial distribution of dielectric permittivity ε(r) decreasing from approximately 2 at the center to 1 at the surface, according to the following relationship:

(1) $$n\left(r\right)=\sqrt{\varepsilon (r)}=\sqrt{2-{\left(\frac{r}{R}\right)}^{2}},$$

where n is the refractive index at point r; ε(r) is the relative dielectric permittivity of the lens material at point r; and r/R is the ratio of the current radial coordinate to the sphere radius.

**Figure 1:**
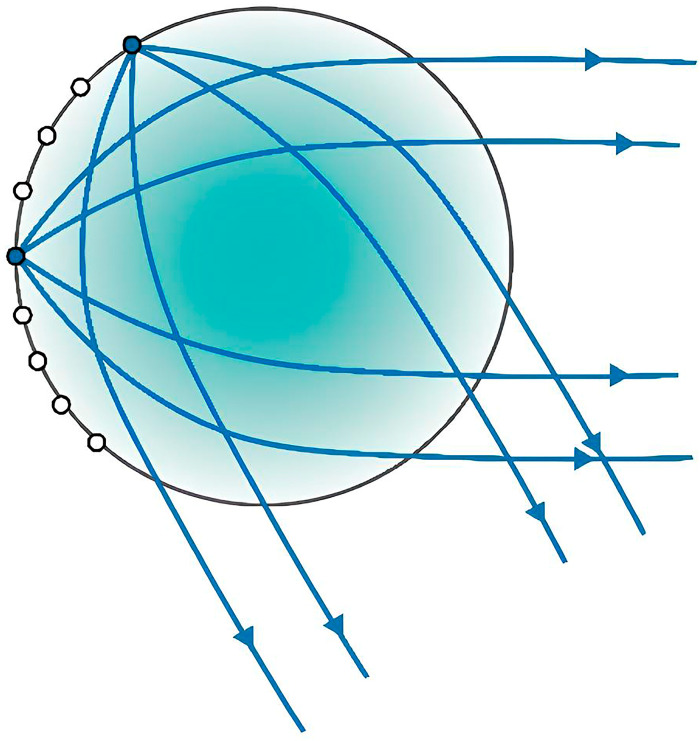
Ray passage in a spherical Luneburg lens.

In the classical Luneburg lens (LL), this condition is implemented via the variation in the lens’s dielectric permittivity from 2 at the center to 1 at the surface. The ray propagation scheme is shown in Figure 1:

The sophisticated technology required to manufacture multilayer lattices with spherical symmetry remains one of the main challenges in the fabrication of Luneburg lens antennas, long constraining progress in this area of antenna engineering.

The practical implementation of a continuous radial gradient of ε presents significant technological difficulties. Typically, the lens is fabricated using a step-approximation approach, in which each layer possesses a constant dielectric permittivity, fabricated from materials with different ε values and assembled as hemispherical shells, by perforating solid dielectric materials [2,3,4]. Due to this discrete construction, the variation in dielectric permittivity along the lens radius occurs stepwise, which reduces the electromagnetic field gain [5].

**Figure 2:**
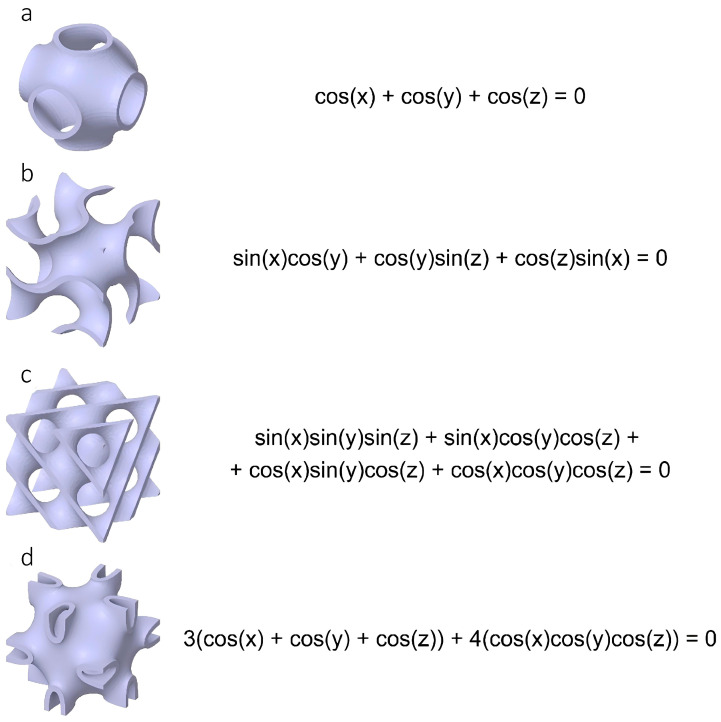
Various shapes of TPMS unit cell lattices: (**a**) Primitive, (**b**) Gyroid, (**c**) Diamond, (**d**) Neovius and their approximation equations, where 0 is the level-set parameter, and x = 2πnX/L, y = 2πnY/L, and z = 2πnZ/L with n is the number of unit cells (i.e., periodicity), L is the size of the structure, and X, Y, and Z are the Cartesian coordinate system.

Additive manufacturing (AM) offers an alternative route for fabricating such gradient dielectric devices, enabling precise control over internal geometry and spatial density [6,7]. A particularly promising approach involves triply periodic minimal surfaces (TPMS)—mathematically defined surfaces with zero mean curvature, three-dimensional periodicity, and minimal surface area. Due to their smooth saddle-shaped topology, TPMS lattices combine isotropic mechanical response, low stress concentration, and tunable porosity, allowing control over dielectric permittivity through architectural design [8,9,10,11]. Figure 2 illustrates several types of TPMS geometries (Primitive, Gyroid, Diamond, Neovius) that form the basis of the proposed design.

**Figure 3:**
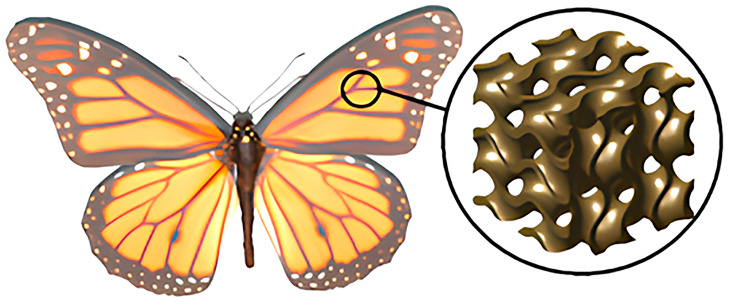
Butterfly wing with hierarchical micro- and nanostructures resembling triply periodic minimal surface architecture. Schematic comparison between natural structure and computationally generated TPMS unit cell.

In natural systems, hierarchical cellular architectures provide gradients of density and refractive index that ensure both lightness and strength [12,13]. For example, butterfly wings exhibit a periodic micro- and nanostructure that closely resembles triply periodic minimal surface (TPMS) geometry, enabling selective light propagation and structural rigidity (Figure 3). These natural principles form the foundation of biomimetic TPMS lattices, where smooth variations in geometry and porosity translate into controllable gradients of mechanical and dielectric properties [14].

By adjusting wall thickness and relative density (RD), TPMS lattices can realize a functional gradient of ε comparable to that required in a Luneburg lens [15]. RD refers to the fraction of the total volume occupied by solid material compared to a fully dense sample made of the same material. This biomimetic principle, inspired by natural cellular architectures with smoothly varying density, allows combining mechanical robustness and electromagnetic tunability in a single structure. Although, owing to recent advances in AM, TPMS-based lattices are increasingly applied as multifunctional solutions for various fields [16], their potential in radio-engineering devices, particularly for gradient-index antenna lenses, remains insufficiently studied.

To address this gap, the present work proposes a TPMS-based additive manufacturing approach to realize a spherical Luneburg lens with a controlled distribution of dielectric permittivity. A tunable ABS–TiO_2_ composite developed for fused-filament fabrication (FFF) was employed, providing variable permittivity and sufficient mechanical strength. In this study, the influence of titanium dioxide content (0%, 20% and 40%) in an ABS plastic matrix on the dielectric and mechanical characteristics of TPMS architectures was systematically investigated. The TiO_2_ concentrations of 0%, 20%, and 40% were chosen to represent pure unfilled ABS (0%), an intermediate filler content with stable extrusion and good dispersion (20%), and the maximum TiO_2_ loading at which a continuous filament suitable for 3D printing can still be produced (40%). The research focused on evaluating the key mechanical performance parameters, including Young’s modulus, yield strength, compressive plateau strength, specific energy absorption (SEA) and toughness, as well as tensile and flexural behavior. Based on the obtained material and structural data, a spherical Luneburg lens prototype was designed using TPMS geometry. The prototype was fabricated by additive manufacturing and experimentally tested to evaluate its radiation pattern (RP), antenna gain, and operational frequency band. The experimental results were compared with numerical simulations, demonstrating stable antenna performance within the 8.0–12.5 GHz range and confirming the feasibility of TPMS-based architectures for multifunctional gradient dielectric devices.

## 2. Materials and Methods

### 2.1. Materials

Since TPMS lattices can only be manufactured by means of additive technologies; consequently, a method was proposed developed for producing a highly filled composite suitable extrusion-based 3D printing. The ABS 2332, a thermoplastic amorphous terpolymer of acrylonitrile, butadiene, and styrene produced by PJSC “SIBUR Holding,” was selected as the basic material for ABS-TiO_2_ composite filament fabrication with mechanical characteristics (flexural strength σ_f_ = 69 MPa, tensile strength σ_t_ = 45 MPa, flexural modulus 2350 MPa), along with titanium dioxide (TiO_2_) in rutile (Crimea TiOx220).

To characterize the initial titanium dioxide powder, a visual analysis of scanning electron microscopy (SEM) micrographs and granulometric analysis and particle size distribution was performed. Elemental composition and the homogeneity of the ceramic filler distribution were examined by scanning electron microscopy (SEM) using a Melytec SM-32 microscope equipped with an energy-dispersive X-ray spectrometer (EDS, Oxford Instruments). A thin carbon layer was deposited on the sample surfaces to dissipate surface charge during analysis.

**Figure 4:**
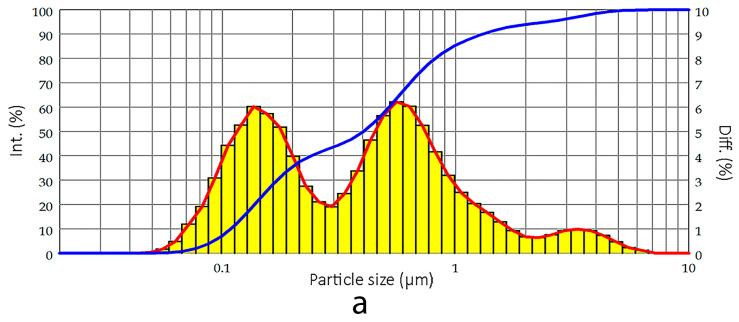

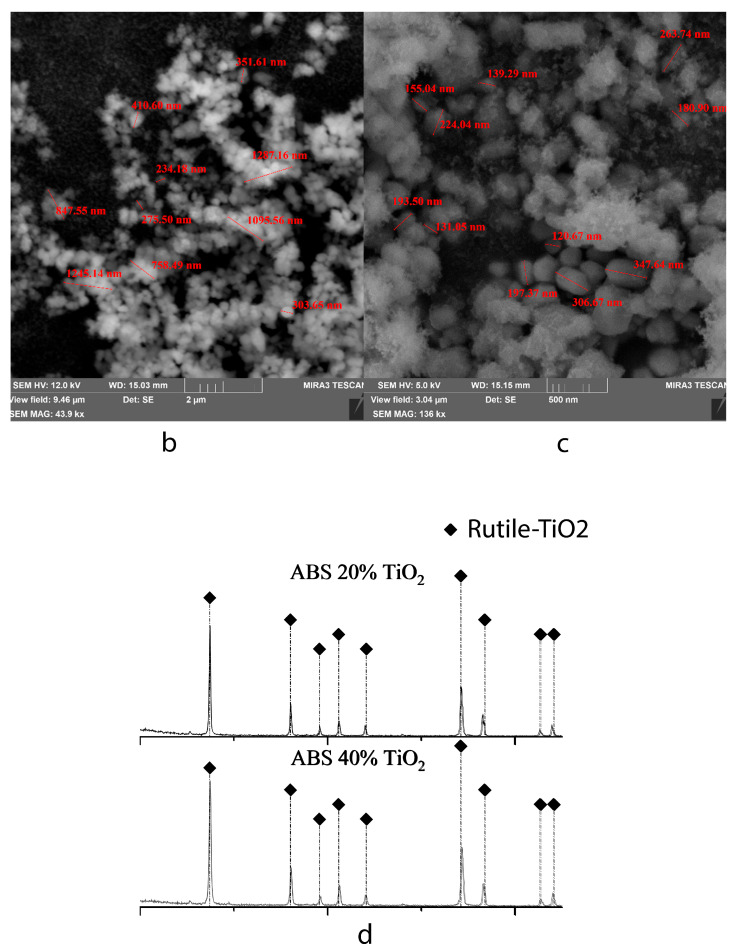
(**a**) Granulometric analysis and particle size distribution of TiO_2_ powder in rutile form, manufactured by the company “Crimean Titan” under the brand name “Crimea TiOx220.” Red line represents the intensity distribution of the particles across different sizes. Blue line represents the cumulative distribution (Diff. %) of the particles. SEM images of rutile TiO_2_ powder (Crimean TITAN Pvt. Ltd., Russia) under the brand name “Crimea TiOx220 at the magnifications (**b**) 43,9 kx (**c**) 136 kx (**d**) XRD patterns of ABS-TiO_2_ composite filaments for 3D-printing with 20 and 40 wt% solid loading.

The titanium dioxide powder used has a bimodal particle size distribution with two peaks at approximately 0.14 μm and 0.52 μm; the average particle size (D50) is 0.404 μm (Table 1, Figure 4a). The granulometric analysis data fully confirm the results obtained by the visual analysis of scanning electron microscope micrographs (Figure 4b,c).

The phase composition of the 3D-printed composite samples was analyzed by X-ray diffraction (XRD) using a DX-2700 diffractometer with Cu Kα radiation. Measurements were performed in continuous scanning mode over a 2θ range of 20–70° at a scan rate of 2°/min. Crystalline phases were identified using the COD and PDF-2 powder diffraction databases. XRD analysis, shown in Figure 4d, indicates that no chemical interaction occurs between the polymer matrix and the ceramic filler during filament fabrication and 3D printing. In the samples containing TiO_2_ (PDF card No. 03-1122 (JCPDS 03-1122)), the phase corresponding to the original component, rutile, is observed, consistent with previously reported data [17].

Titanium dioxide was employed as the filler due to the strong dielectric behavior induced by relaxation polarization under standard temperature conditions (ε = 15–170, tan δ = 0.0016 at 1 MHz) [18,19], which is essential for lens applications. Introducing TiO_2_ into a polymer matrix has been proved to significantly increase the dielectric permittivity of the composite [20], thereby enabling the fabrication of highly porous lattices with the required dielectric properties. 

### 2.2. Preparation of ABS-TiO_2_ Composite Filament

For the fabrication of ABS-TiO2 composite filament, a Coperion ZSK 18 (Coperion GmbH, Stuttgart, Germany) twin-screw compounder setup was employed, featuring co-rotating screws, a 2 mm die, and a downstream pulling system to produce filaments with a diameter of 1.75 mm. The processing temperatures were in the range of 180–200 °C. The polymer was fed into the compounder via a loss-in-weight feeder, melted, and subsequently mixed with the ceramic filler, which was introduced using a similar dosing system. The incorporated filler was thoroughly dispersed in the compounder’s barrel through intermeshing screw elements with a 0.1 mm clearance. The resulting mixture was then extruded through the die and cooled in a water bath to solidify the filament.

### 2.3. Preparation of FFF Samples

The technological parameters of 3D printing were optimized for the developed composite materials. Printing was performed by fused deposition modeling on an “Artillery Sidewinder X1” 3D printer with a nozzle diameter of 0.4 mm, an extruder temperature of 240 °C, a bed temperature of 90 °C, and a printing speed of 60 mm/s.

In this work, ABS-based samples were produced with different TiO_2_ weight fractions: 0, 20, and 40. The maximum achievable filler content was 40%, since materials with a higher TiO_2_ concentration manifested spontaneous fracture, making 3D printing infeasible. The maximum TiO_2_ loading (40 wt.%) was determined experimentally, as higher concentrations caused filament cracking and extrusion instability, preventing continuous 3D printing [21]. All TPMS specimens were printed using a 0.4 mm nozzle and a layer height of 0.2 mm. The build orientation was vertical, with the Z-axis corresponding to the build direction. The raster pattern alternated by ±45° between adjacent layers. Compression tests were performed along the material deposition direction (100) of the TPMS lattice. 

The specimens employed in this study were fabricated from 3 materials: ABS 2332 by PJSC “SIBUR Holding, without TiO_2_, and ABS-TiO_2_ composites with 20 wt% and 40 wt% TiO_2_ filler (Crimea TiOx 220). For the fabrication of the Luneburg lens prototype, unfilled ABS 2332 plastic was used, as this material ensured stable extrusion and defect-free printing during the optimization of the complex TPMS geometry. The use of the unfilled material at this stage allowed us to refine the printing parameters and verify the manufacturability of the spherical TPMS-based architecture, whose integration into the Luneburg lens represents a novel and previously unexplored approach in radio engineering design. In future work, the developed ABS–TiO_2_ composite will be employed for TPMS-based Luneburg lens fabrication to achieve the required dielectric permittivity while maintaining reduced weight and structural integrity. The lens diameter was set to 192 mm, ensuring functionality within the 8–12 GHz frequency band.

### 2.4. Development of Biomimetic Lattices

The geometry of the lattices was based on the TPMS, which exhibit relatively high mechanical properties among cellular materials [22]. To develop gradient lattices, the experiments focused on selecting the most reproducible TPMS lattices that did not contain overhanging elements, the latter typically leading to defects during fabrication.

**Figure 5:**
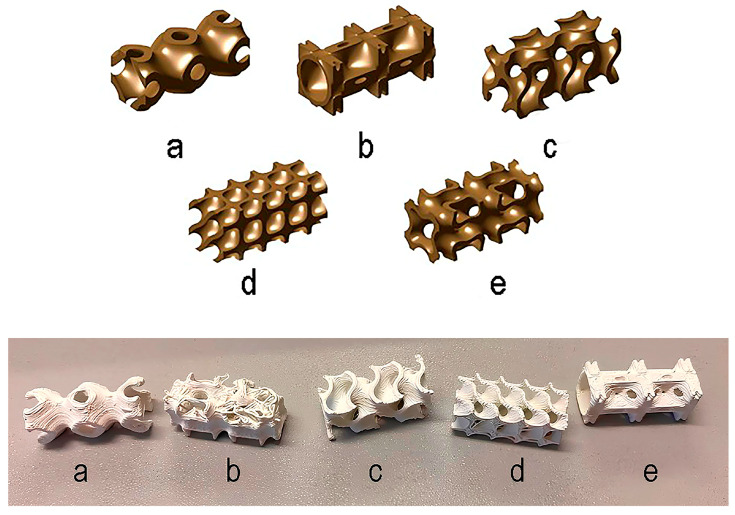
Designed lattices selected for printing and printed TPMS samples from an ABS composite filled with 40 wt% TiO_2_ content. samples of different lattices: (**a**) Primitive, (**b**) IWP, (**c**) Gyroid, (**d**) Diamond, (**e**) Neovius.

Five TPMS lattices were selected, modeled using the “MS Lattice” software [23] for different sheet-based TPMS lattices, namely; Diamond, I-WP, Gyroid, Neovius, Primitive (Figure 5). The samples were parallelepipeds with dimensions of 23 mm × 10 mm × 10 mm and were printed from an ABS composite filled with 0, 20 and 40 wt% TiO_2_ content. The material with the highest filler content was used, as it exhibited the poorest print quality (Figure 5). This choice allowed us to illustrate the effect of high TiO_2_ concentration on extrusion stability and surface quality of the printed parts.

Mechanical tests were conducted with the results based on the average of 3 specimens each. Experimental data analysis was performed using the “Origin” software [24].

A 3D model of the spherical TPMS prototype was created using «Meshmixer» (Autodesk, Inc., San Francisco, CA, USA) [25]. The ABS-TiO_2_ composite filament featured defects during fabrication: voids, cracks, over-extrusion. The filled composite requires further optimization of printing parameters. Therefore, to eliminate the significant influence of 3D-printing quality on the subsequent results, we used ABS 2332 material without TiO_2_ content. Nevertheless, the high permittivity of titanium dioxide in the ABS-TiO_2_ composite filament reduces the RD of parts. As a preliminary control, RD was verified by mass: the weight of a solid sample was compared with that of a TPMS-structured sample. The density of the base material (ABS material) was taken as 1.08 g/cm^3^. The obtained values of RD were in agreement with the designed ones within 3%, as illustrated by the example of a G-TPMS sample without TiO_2_ content with RD = 30%.

In future work, the developed ABS-TiO_2_ composite filament material will be employed to decrease the overall weight of the device.

### 2.5. Mechanical Behavior

The assessment of the mechanical performance of TPMS-based materials is essential to ensure the structural reliability of the Luneburg lens throughout its service life. Although the lens itself functions as a passive electromagnetic element, its mechanical stability directly influences the accuracy of the dielectric gradient and, consequently, the focusing quality and radiation efficiency. Antenna lens may be stationary or integrated into primary load-carrying composite skins—such as aircraft fuselage panels, wings, or other aerodynamic surfaces—they experience the same mechanical environment as the supporting structure. The dominant sources of in-service mechanical loads include tension, compression, bending stresses, as well as sudden impacts and vibrations. These loads induce global deformation and strain accumulation in the antenna’s substrate material [26]. To determine the TPMS geometry with the best mechanical performance, physical and mechanical tests were conducted on ABS-based samples with different titanium dioxide (TiO_2_) contents. The compression test specimens were cubes with an edge length of 30 mm and various TPMS lattices. In this work, we assume that cubic TPMS architectures behave similarly to spherical architectures. The tests were performed in accordance with [27].

Flexural test specimens were prepared as beams (sample dimensions 80 mm × 10 mm × 4 mm), and tensile test specimens were dog-bone type (Type 1A). The specimen dimensions were selected in accordance with standard [28]. The tests were performed on a universal tabletop testing machine “Metotest REM-50-A-1-1” at a loading rate of 5 mm/min.

The authors of [29] demonstrated that the mechanical properties of sheet-based TPMS cellular materials are strongly dependent on the presence of defects, in contrast to their fully filled ligament-based counterparts. However, since the wall thickness in their case was relatively large (0.4 mm), we believe that the defect content might have been underestimated.

To obtain specific strength values the mechanical test results were adjusted relative to the sample density. The mass of each specimen was determined using “Ohaus Pioneer PA–214C” analytical balances. The beam volume was determined geometrically from the overall dimensions using a caliper “ShTsTs-1-150-0.01,” while the volume of the dog-bone specimens was calculated from 3D models in SolidWorks software SP5.0 (Dassault Systèmes inc., France) [30].

### 2.6. Dielectric Characterization, Measurement and Simulation of Radiation Properties

In our work, the dielectric permittivity was measured for samples with different RD and TiO_2_ contents. The antenna characteristics, however, were evaluated only for the prototype fabricated from ABS 2332 material without TiO_2_ content

To characterize the dielectric response, the composite was printed into fully dense (RD = 100%) samples with different TiO_2_ content. The test pieces were rectangular prisms with dimensions of 23 mm × 10 mm × 10 mm. The dielectric properties of the developed composite and TPMS lattice samples were characterized using a short-circuited rectangular waveguide method. Measurements were performed at JSC “NII Vector” employing a P1-28 transmission line setup operating in the X-band (8–18 GHz). The experimental procedure followed the standard waveguide reflection technique for materials completely filling the waveguide cross-section. Initially, the reference short-circuit position was determined by recording the standing-wave minima in the empty waveguide. Subsequently, the dielectric specimen was inserted into the waveguide, and the new minima positions were registered. The observed shift in the standing-wave pattern was used to calculate the complex dielectric permittivity of the sample. For each material composition, three independent measurements were conducted to ensure reproducibility, with the average value reported. The measurement uncertainty did not exceed ±3%. For the solid (100% infill) ABS samples containing 40 wt% TiO_2_, the relative dielectric permittivity) in the 8–18 GHz frequency range was determined as ε = 3.95, while the unfilled ABS sample exhibited ε = 2.6. The same technique was applied to TPMS structures with varying relative density (RD) to establish the dependence ε(RD) used for the lens design in accordance with [31]. 

Radiation pattern measurements were performed using the far-field method. A horn radio signal emitter operating in the 8–18 GHz range was positioned at the focal point of the lens. A P6-23M horn antenna served as the receiving antenna. The measurements were performed with horizontal polarization, where the electric field vector of the electromagnetic wave was oriented parallel to the Earth’s surface. Radiation pattern (RP) characterization of the lens prototype was conducted in a laboratory environment using scattering-absorbing panels instead of a full anechoic chamber.

The transmitting and receiving horn antennas were positioned at a separation distance of 5 m, ensuring far-field conditions within the operating frequency range. The antenna under test (AUT) was mounted on a motorized rotation stage and scanned in azimuth with a step size of 1°, while the frequency step was 10 MHz. Four azimuthal radiation pattern cuts were recorded at different rotational orientations of the lens (0°, 90°, 180°, 270°). The antenna gain was determined using the classical three-antenna method.

Absolute gain calibration, polarization purity measurements, and detailed uncertainty analysis were not included at this stage due to the preliminary nature of the experimental setup. These procedures, along with measurements in a certified anechoic chamber with a defined quiet zone and standardized calibration methods, are planned for future studies.

**Figure 6:**
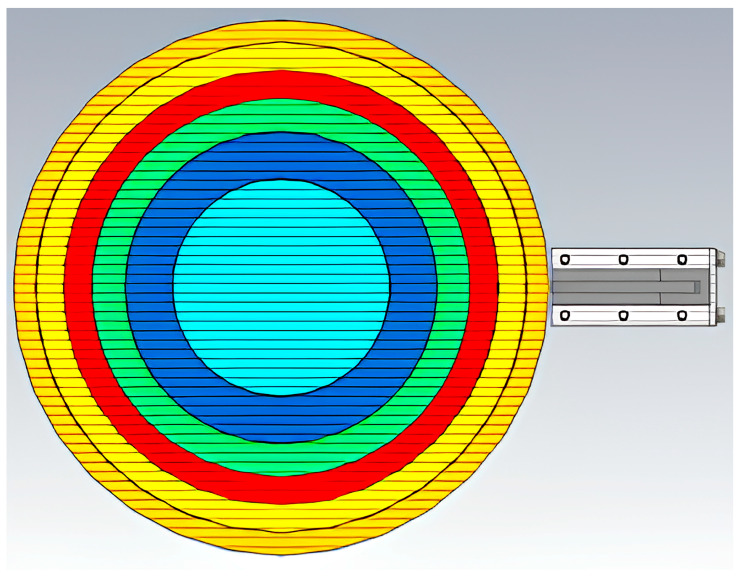
Cross-section of the ideal spherical Luneburg lens model with a radio signal emitter. Different colors in the figure schematically represent different values of dielectric permittivity. Light blue corresponds to ε = 1.93, blue to ε = 1.77, green to ε = 1.61, red to ε = 1.46, yellow to ε = 1.31, and orange to ε = 1.16.”.

Numerical electromagnetic simulations of the reflection pattern, along with the calculation of RP and antenna gain were performed in CST Studio 2021. To evaluate the measured RP and gain of the spherical TPMS prototype, the latter was compared with an ideal model of a Luneburg lens (Figure 6).

## 3. Results and Discussion

### 3.1. Effect of Topology on Composite Printability

The reproducibility of the shape for TPMS lattices was assessed by fused filament fabrication (FFF) printing, with quality control based on the deviation between the designed and fabricated samples. The printing results showed the fewest defects (surface roughness, voids, cracks, etc.) in the following lattices: Gyroid, Diamond and Neovius (Section 2.4, Figure 5). Samples (a) and (b) in Figure 5 exhibited printing defects and were therefore excluded from further consideration.

The Gyroid, Diamond and Neovius showed the best manufacturability among the tested TPMS lattices with different TiO_2_ contents. The Primitive and IWP samples exhibited significant defects, most likely due to the presence of overhanging structural elements that are difficult to reproduce when printing small-sized specimens. Therefore, the latter lattices were excluded from further consideration.

The lattice types of the examined Gyroid topology promised to be the most promising, combining minimal printing defects with a wide range of achievable dielectric permittivity values. The choice of geometry is discussed in more detail in Section 3.2. In contrast, for the Neovius and Diamond, low permittivity values were not possible to achieve, since the lattices failed to reproduce at low filling fractions. Consequently, these TPMS lattices were also excluded from further consideration.

### 3.2. Effect of Topology on Mechanical Behavior

**Figure 7:**
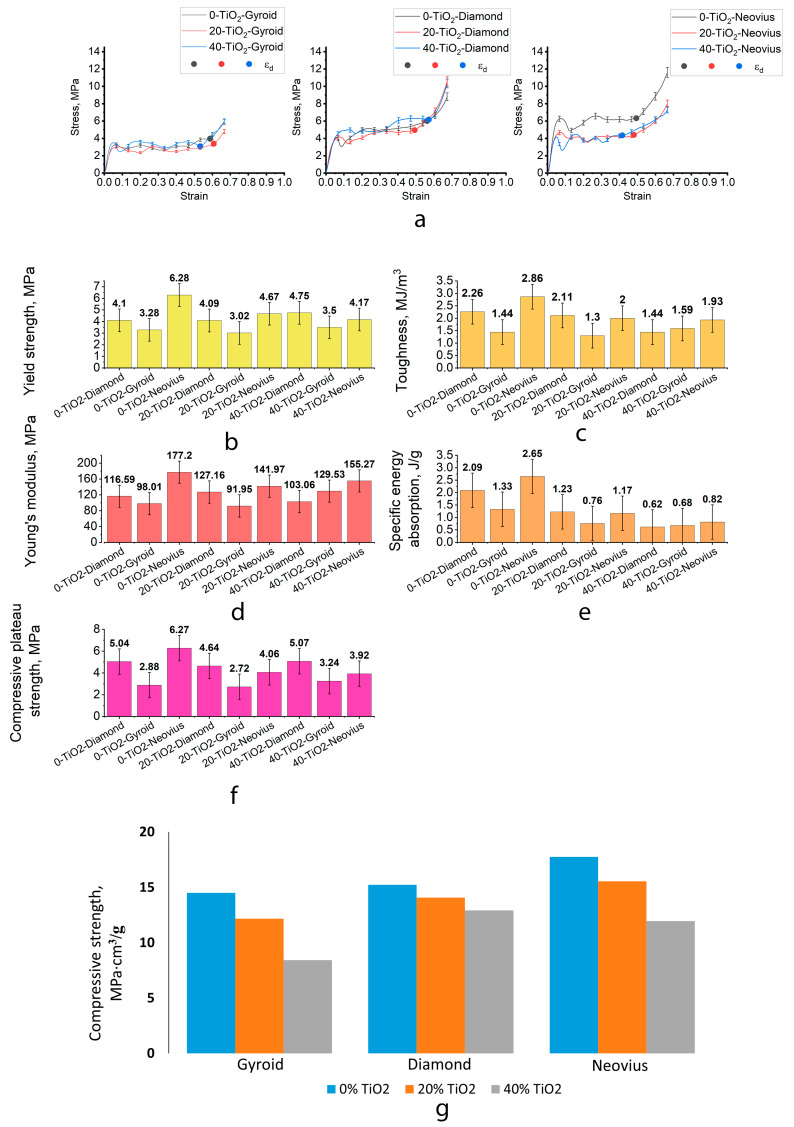
(**a**) Experimental deformation curves of samples with different types of TPMS lattices were fabricated using either the pure ABS material or the ABS-TiO_2_ composite material. Mechanical properties of samples with different types of sheet-based TPMS lattices fabricated using either pure ABS or the ABS/TiO_2_ composite material: (**b**) Yield Strength, (**c**) Toughness, (**d**) Young’s Modulus, (**e**) SEA, (**f**) Compressive Plateau Strength, and (**g**) Histogram of the specific strengths of samples with different types of sheet-based TPMS fabricated using either the pure ABS material or the ABS-TiO_2_ composite material.

The results of mechanical tests revealed the following characteristics: Young’s modulus, yield strength, compressive plateau strength, toughness and SEA of the TPMS polymeric lattices (Figure 7b–f), tensile strength and flexural strength (Table 2), as well as their specific values. The densification strain was defined as the point of onset of densification and was extracted from the intersection of tangent lines for the plateau region (characterized by constant mean stress) and the densification region (characterized by a rapid and linear increase in stress). The mass of the samples is presented in Table 3.

As evidenced by the obtained mechanical test results, the 3D-printed TPMS specimens fabricated from ABS without TiO_2_ exhibited the highest values of the key mechanical parameters—including Young’s modulus, yield strength, compressive plateau strength, toughness and SEA—compared with the other lattices with ABS–TiO_2_ composite material (Figure 7b–f). Moreover, the addition of titanium dioxide reduced the specific tensile and flexural strength values (Table 2). However, the differences were not pronounced, indicating that filled specimens may also be regarded as appropriate for the fabrication of radio engineering components while still maintaining adequate mechanical performance.

This indicates that the addition of TiO_2_ slightly compromises the mechanical performance but does not critically affect structural stability, allowing the use of filled composites where higher permittivity is required. According to the mechanical property data obtained (Figure 7g), Neovius topology exhibited the greatest compressive strength, whereas the lowest values were recorded for Gyroid.

The stress–strain behavior of the TPMS-based lattices (Figure 7a) revealed a stepwise energy absorption mechanism. A similar cellular material’s compressive stress–strain curve was observed [32]. The stress–strain response shows an initial linear region, followed by a plateau that continues until densification. This plateau is characterized by a stable stress–strain behavior without significant stress variations. Such behavior indicates that the printed TPMS architectures preserve their energy absorption capability under compressive loading. In particular, the Gyroid geometry maintains mechanical stability even at lower weight, enabling its application in lightweight gradient structures where structural integrity and controlled porosity must coexist. This progressive cell collapse mechanism explains the high energy absorption capacity and confirms the suitability of TPMS architectures for vibration-damping or load-bearing antenna substrates.

For all investigated lattices, an increase in TiO_2_ content resulted in a systematic reduction in specific strength. This tendency reflects a fundamental change in the deformation mechanism of the composite. The introduction of TiO_2_ particles leads to the formation of interfacial regions with reduced adhesion strength between the filler and the polymer matrix. As the filler concentration increases, these interfaces act as preferential sites for stress localization and crack initiation. The ensuing phase segregation into TiO_2_-rich and polymer-rich domains enhances microstructural heterogeneity, promoting the development of local stress gradients and premature failure.

Consequently, the material response transitions from ductile plastic deformation, characteristic of unfilled ABS, to a brittle fracture regime dominated by interfacial debonding and particle-induced crack propagation. This behavior highlights the critical role of filler dispersion and interfacial compatibility in balancing the dielectric enhancement and mechanical integrity of TiO_2_-modified polymer lattices. Therefore, the optimal filler fraction should be determined by balancing these opposing effects to maintain sufficient mechanical integrity for large-scale 3D-printed antenna components.

**Figure 8:**
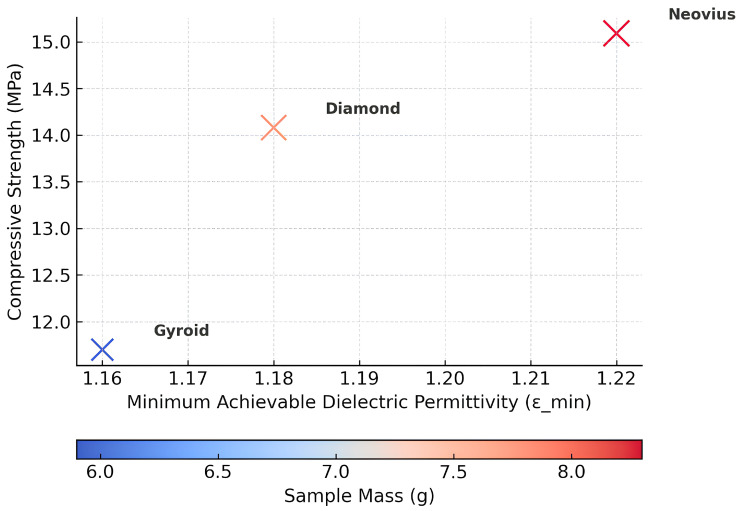
Structure–property selection map identifying the optimal TPMS geometry based on permittivity, strength, and mass criteria.

For the fabrication of a Luneburg lens, it is necessary to achieve a low dielectric permittivity in the outer layer by employing a low relative density structure. Therefore, an important property is the ability of a lattice with a given architecture to be printed reliably over a wide RD range with good manufacturability. Among the investigated TPMS architectures, the Neovius geometry exhibited the highest compressive strength. However, the highest manufacturability for radio engineering applications among the studied TPMS structures was demonstrated by the Gyroid, which allows the creation of various Luneburg lens layers across a wide RD range while maintaining acceptable mechanical properties and low weight (Table 3). Among the studied structures, the Gyroid sample exhibited the lowest mass (5.9 g), while the Diamond and Neovius structures were heavier, with masses of 7.8 g and 8.3 g, respectively. The reduced mass of the Gyroid geometry results from its more open and continuous minimal-surface architecture, which provides lower relative density while maintaining structural stability. This characteristic makes the Gyroid topology advantageous for lightweight applications and gradient dielectric components, confirming its suitability for the fabrication of Luneburg lens layers where both low density and sufficient mechanical integrity are required (Figure 8).

Therefore, the ability of Gyroid TPMS architecture to maintain mechanical integrity across a wide RD range and low weight is essential for practical implementation in gradient dielectric devices such as Luneburg lenses.

### 3.3. Effect of Topology on Dielectric Permittivity

**Figure 9:**
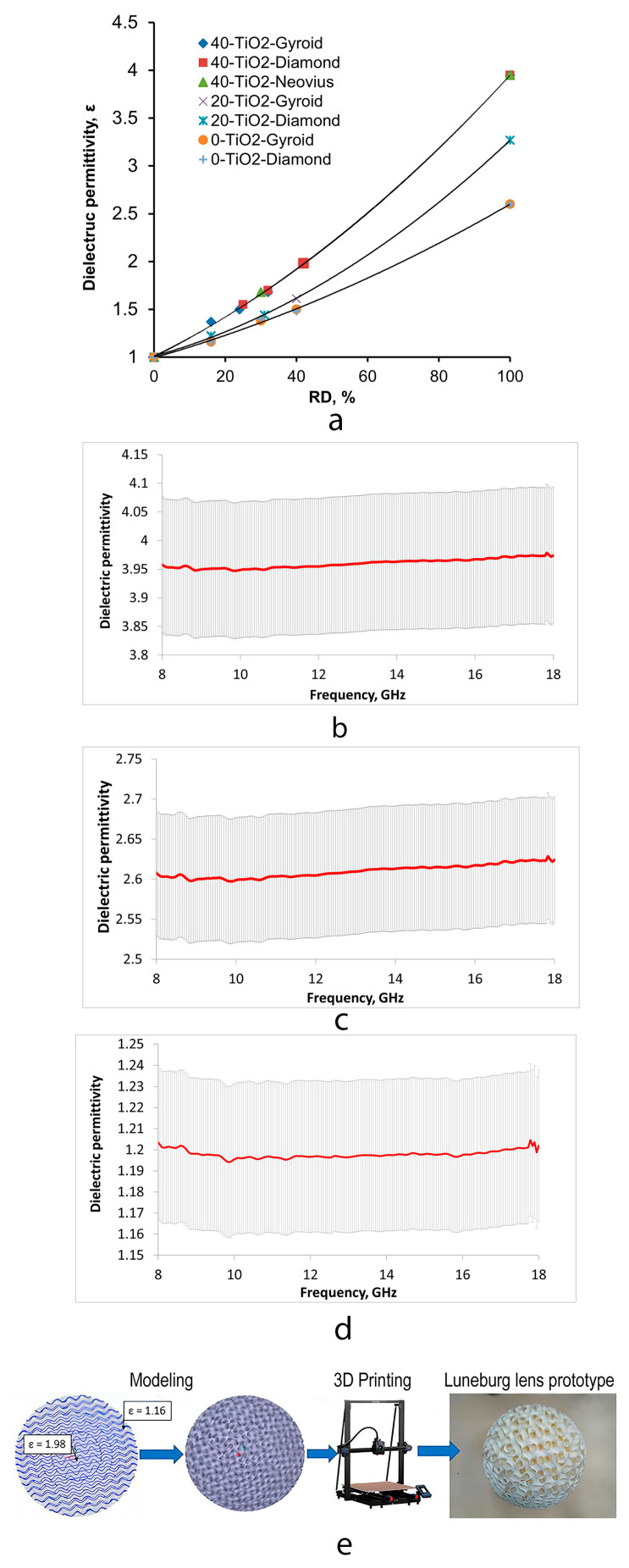
(**a**) Dependence of the dielectric permittivity ε on the RD for the lattices with different TPMS architectures fabricated using either pure ABS or the ABS-TiO_2_ composite material. Measured dielectric permittivity (ε) in the 8–18 GHz frequency range for the (**b**) dense ABS sample with 40 wt.% TiO_2_, the (**c**) unfilled dense ABS sample (0 wt.% TiO_2_), and the (**d**) TPMS-based lattice structure with a relative density of 16%. The variation in the permittivity of samples changes is less than 3%. (**e**) General scheme of the 3D printed antennas fabrication based on TPMS lattices. The red arrow indicates the X-axis, and the green arrow indicates the Y-axis of the 3D models.

The dielectric property measurements revealed a dependence (Figure 9a) between the RD and the dielectric permittivity for the lattices with different TPMS architectures fabricated using either pure ABS or the ABS-TiO_2_ composite material. Dielectric measurements showed that dielectric permittivity ε decreases with reduced RD. The obtained dependence of the dielectric permittivity ε on the RD for the lattices with different TPMS architectures fabricated using either pure ABS or the ABS-TiO_2_ composite material is described by the Lichtenecker formula for composite materials [33]:

(2) $$\varepsilon^{k}=\varphi \cdot \varepsilon_{1}^{k}+\left(1-\varphi \right)\cdot \varepsilon_{2}^{k},$$

where ε_1_ is the dielectric permittivity of medium 1 (material), ε_2_ is the dielectric permittivity of medium 2 (air), φ is the space filling fraction of the polymer, and k = 0.45694.

The coefficient k was obtained by polynomial fitting, where k is the isotropic coefficient. It can take up the values of [−1; 1]; the values of |k| = 1 correspond to the highest anisotropy, and the value of k = 0 correspond to the ideal geometric isotropy [34]. Our value for of k = 0.45694 depicts the average anisotropy, halfway short of the ideal isotropy.

In effective medium theory [35], the effective permittivity of a two-phase dielectric composite must lie between the classical Wiener bounds, which correspond to the two extreme field configurations: perfectly parallel layers (upper bound) and perfectly series layers (lower bound). These bounds are exact only for ordered, layered systems. In practical polymer–ceramic or polymer–air composites the microstructure is neither purely series nor purely parallel, but consists of a random network of displacement-current paths that sample both configurations. Therefore, an effective-medium approximation must interpolate between the two Wiener limits.

One convenient way to construct such an interpolation is to perform the averaging not on ε itself, but on its logarithm. The logarithmic (Lichtenecker) mixing law (Equation) assumes that each phase contributes to the effective response proportionally to its volume fraction φᵢ, while the complex, interpenetrating connectivity of the phases is represented by the multiplicative (geometric) rather than additive averaging:

(3) $$\log\varepsilon_{eff}=\varphi_{1}\cdot \log\varepsilon_{1}+\varphi_{2}\cdot \log\varepsilon_{2},$$

where $\varepsilon_{eff}$ is the effective permittivity of the composite, ε_1_ and ε_2_ are the permittivities of phases 1 and 2, φ_1_ and φ_2_ are their volume fractions (φ_1_ + φ_2_ = 1).

From the viewpoint of effective medium theory, the electric field in random isotropic composites is distributed non-uniformly: in some regions, the phases are connected mainly in parallel, while in others in series. Thus, the displacement current partially passes through areas where the field behaves as in the parallel configuration and partially through those resembling the series configuration. As a result, the overall (effective) dielectric permittivity of the material takes an intermediate value, which is close to the geometric mean of the permittivity of the individual phases. In a more general form, the rule is written as (Equation (2)).

As shown in the study [36], the Lichtenecker low provides a good approximation of the experimental data for polymer–ceramic composites, such as those based on barium titanate, which confirms its applicability within the effective medium approximations for statistically isotropic dielectric mixtures.

In TPMS-based structures, variations in effective ε are primarily associated with the amount of air in the pores rather than with changes in material composition. As RD decreases, the fraction of air voids (ε ≈ 1) increases, resulting in a reduction in the effective dielectric permittivity of the structure [37].

**Figure 10:**
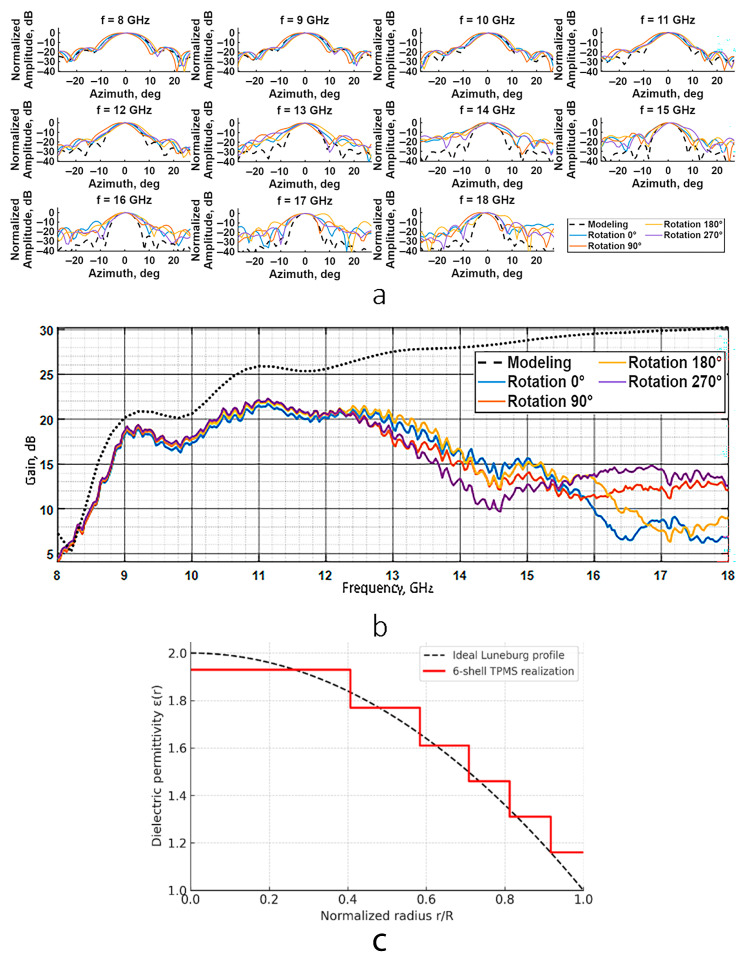
(**a**) Azimuthal cross-sections of the radiation patterns at frequency 8–18 GHz for Luneburg lens prototype based on TPMS lattices. (**b**) Luneburg lens prototype based on TPMS lattices gain plotted as a function of frequency. (**c**) The approximation of the Luneburg law in the fabricated spherical lens prototype.

Accordingly, the results presented in Figure 10a, which are approximated and described by effective medium mixing laws (in this work, the Lichtenecker formula was employed), can be explained by the fact that as the relative density (RD) of the structure increases, the fraction of the composite phase with higher permittivity becomes larger, thereby contributing more significantly to the overall dielectric response. Consequently, the effective permittivity ($\varepsilon_{eff}$) of the TPMS structure increases in a consistent and predictable manner with increasing RD.

### 3.4. Fully 3D Printed Abs Based Antenna with TPMS Lattices Topology

To select the geometric parameters of the layers we used [38]. This work presents the recommendations for the design of multilayer Luneburg lenses (LL). Various optimization tasks are described in detail, aimed at minimizing three different norms of deviation between the ideal and reconstructed relative dielectric permittivity, depending on the thickness and permittivity of each shell. In this study, the influence of the number of layers in a spherical Luneburg lens on its antenna characteristics were analyzed, including sidelobe levels and gain, for various lens diameters, with the aim of optimizing these parameters. Based on the thickness and dielectric permittivity proposed in this work, as well as Equation (2), we designed the required relative densities (RD), as well as the dimensions and thicknesses of the hemispheres for the Luneburg lens prototype.

Modeling of the Luneburg lens prototype was carried out using the Meshmixer software (Autodesk). Separate hemispheres with Gyroid-type TPMS geometry were designed, with the RD of each layer adjusted to achieve the target dielectric permittivity values specified in Table 4. The RD represents the ratio of the solid material volume to the total structure volume: as RD increases, dielectric permittivity (ε) rises according to Equation (2). Subsequently, six designed hemispheres with different RD values, ranging from 77% to 16%, were sequentially combined into a single three-dimensional model of the lens, forming a continuous radial gradient of dielectric permittivity (Figure 10c). It should be noted that the connections between adjacent hemispherical layers were incorporated at the design stage, which allowed the lens to be printed as a single monolithic structure. The resulting 3D model was exported into a slicing-compatible format and processed in “Anycubic Slicer.” The control code (g-code) was generated using the optimized printing parameters. Manufacturing was carried out on an “Anycubic Kobra 2 MAX” 3D printer (Figure 9e).

The parameters of layers are presented in Table 4. The outer layer target (ε = 1.16) (Table 4) for the Luneburg lens was obtained using Gyroid RD = 16%. The central (№1 in Table 4) layer target (ε = 2) was achieved with RD ≈ 42% Gyroid. The resulting scheme of the layer configuration for the spherical Luneburg lens prototype is shown in Figure 10c.

To quantitatively assess how much six-layer profile deviates from the ideal Luneburg lens profile (Equation (1)), we calculated the deviation using the L∞ norm. To quantitatively assess how much six-layer profile deviates from the ideal Luneburg lens profile ε(r) = 2 − (r/R)^2^, we calculated the deviation using the L∞ norm:

(4) $$L\infty =max\left|\varepsilon_{real}(r_{i})-\varepsilon_{ideal}(r_{i})\right|,\;i=1..N,$$

where

$\varepsilon_{real}(r_{i})$—the realized permittivity value for the i-th radial layer of the lens;
$\varepsilon_{ideal}(r_{i})$—the ideal Luneburg permittivity value at the same radius ri;
$r_{i}$—the radius of the i-th layer;
N—the number of discrete layers (in this case, N = 6);
L∞—the maximum (worst-case) deviation between the realized and ideal profiles.

In the present six-shell approximation, the maximum deviation from the ideal Luneburg profile ε(r) = 2 − (r/R)^2^ is L∞ = 0.16 (at the outer shell).

The radiation characteristics of the fabricated prototype were subsequently examined and compared with the results of finite element method (FEM) simulations (Figure 10a,b).

The normalized radiation patterns in the 8–12 GHz range (Figure 10a) showed strong qualitative agreement with the computational model. This agreement verifies that the printed TPMS-based layers reproduce the designed dielectric gradient with sufficient accuracy in the intended frequency band.

At higher frequencies (13–18 GHz), however, deviations become evident, sidelobe levels increase to −7 dB, and the main lobe begins to distort, especially at 180° rotation.

The frequency dependence of the antenna gain is presented in (Figure 10b). In the 8.0–12.5 GHz range, the prototype gain demonstrates qualitative agreement with the model within this frequency band, while beyond 13 GHz it decreases.

This deviation indicates that the actual dielectric distribution of the printed lens departs from the continuous ideal profile, resulting in phase errors across the aperture. The mismatch between adjacent layers leads to partial reflections and local distortion of the wavefront, which manifests as increased sidelobe levels and reduced focusing efficiency. Consequently, the antenna gain decreases, since a portion of the radiated energy is redistributed from the main lobe into the sidelobes. In the present case, the maximum deviation L∞ = 0.16 at the outer shell primarily affects the periphery of the aperture, where even small phase mismatches cause noticeable degradation of directivity and an increase in the sidelobe level at frequencies above 12.5 GHz.

Thus, the fabricated Luneburg lens prototype from TPMS lattices demonstrates correct operation in the 8.0–12.5 GHz frequency range.

## 4. Conclusions

In this study, a ABS-TiO_2_ composite filament with 0–40 wt% solid loading was developed to increase dielectric permittivity. The mechanical and dielectric properties of several TPMS lattice structures (Gyroid, Diamond, Neovius, IWP, and Primitive) were studied. Among them, the Gyroid lattice was identified as the most promising topology that combines minimal printing defects with a wide range of achievable dielectric permittivity values. The relationship between dielectric permittivity and relative density (RD) in TPMS lattice structures was found to follow Lichtenecker’s law. A prototype of the Luneburg lens with a TPMS-based geometry and radial distribution of dielectric permittivity was fabricated using this relationship.

The radiation characteristics and the frequency dependence of the antenna gain of the fabricated prototype were subsequently examined. In the 8.0–12.5 GHz range, the prototype’s gain and the normalized radiation patterns demonstrate qualitative agreement with the numerical simulations within this frequency band. At higher frequencies (13–18 GHz), distortions of the main lobe were observed, particularly at a rotation angle of 180°, which may be attributed to structural heterogeneities or internal reflections caused by deviations from the theoretical permittivity profile. Therefore, the TPMS lattices studied here deliver mechanical robustness and functional properties for lens antenna application.

## Figures and Tables

**Table 1:** Fractional composition of the TiO_2_ powder. D10 is the particle diameter below which 10% of the total volume of particles is found, representing the fine fraction of the distribution. D50 is the median particle size, meaning that 50% of the particles are smaller and 50% are larger than this value. D90 is the particle diameter below which 90% of the total volume of particles is found.

	D10, µm	D50, µm	D 90, µm
TiO_2_	0.109	0.404	1.303

**Table 2:** Results of mechanical tests of 3D-printed control samples with different types of sheet-based TPMS fabricated using either the pure ABS material or the ABS/TiO_2_ composite material.

TiO_2_ Content Strength σ	0%	20%	40%
σ_t_, MPa	29.8 ± 1.1	27.1 ± 0.9	26.5 ± 1.0
σ_t.s_., MPa ∙ cm^3^/g	32.8 ± 1.4	25.2 ± 1.0	19.5 ± 0.8
σ_f_, MPa	48.7 ± 2.0	47.3 ± 1.7	44.1 ± 1.8
σ_f.s._, MPa ∙ cm^3^/g	53.3 ± 2.5	44.0 ± 1.8	34.0 ± 1.6

(σ_t_)—tensile strength; (σ_t.s_)—specific tensile strength; (σ_f_)—flexural strength (σ_f.s_)—specific flexural strength.

**Table 3:** Mass of compression samples with different types of sheet-based TPMSs were fabricated using the pure ABS material.

TPMS Geometry	Mass, g
G-TPMS	5.9
D-TPMS	7.8
N-TPMS	8.3

**Table 4:** Parameters of the spherical prototype based on the TPMS lattice.

Layer Number	Diameter of Layer, mm	RD of Layer, %	ε
1	78	77	1.93
2	112	64	1.77
3	136	50	1.61
4	156	39	1.46
5	176	27	1.31
6	192	16	1.16

## Data Availability

The dataset is available on request from the authors.

## Abbreviations

The following abbreviations are used in this manuscript:

TPMSs	Triply Periodic Minimal Surfaces
PRs	Radiation Patterns
SEM	Scanning Electron Microscopy
AM	Additive Manufacturing
FFF	Fused Filament Fabrication
ABS	Acrylonitrile Butadiene Styrene
